# Discrepancies in vegetation phenology trends and shift patterns in different climatic zones in middle and eastern Eurasia between 1982 and 2015

**DOI:** 10.1002/ece3.5408

**Published:** 2019-07-12

**Authors:** Yaobin Li, Yuandong Zhang, Fengxue Gu, Shirong Liu

**Affiliations:** ^1^ Key Laboratory of Forest Ecology and Environment, State Forestry and Grassland Administration, Institute of Forest Ecology, Environment and Protection Chinese Academy of Forestry Beijing China; ^2^ Key Laboratory of Dryland Agriculture, Ministry of Agriculture, Institute of Environment and Sustainable Development in Agriculture Chinese Academy of Agricultural Sciences Beijing China

**Keywords:** climatic zone, NDVI, phenology, shift pattern, the middle and eastern Eurasia region

## Abstract

Changes in vegetation phenology directly reflect the response of vegetation growth to climate change. In this study, using the Normalized Difference Vegetation Index dataset from 1982 to 2015, we extracted start date of vegetation growing season (SOS), end date of vegetation growing season (EOS), and length of vegetation growing season (LOS) in the middle and eastern Eurasia region and evaluated linear trends in SOS, EOS, and LOS for the entire study area, as well as for four climatic zones. The results show that the LOS has significantly increased by 0.27 days/year, mostly due to a significantly advanced SOS (−0.20 days/year) and a slightly delayed EOS (0.07 days/year) over the entire study area from 1982 to 2015. The vegetation phenology trends in the four climatic zones are not continuous throughout the 34‐year period. Furthermore, discrepancies in the shifting patterns of vegetation phenology trend existed among different climatic zones. Turning points (TP) of SOS trends in the Cold zone, Temperate zone, and Tibetan Plateau zone occurred in the mid‐ or late 1990s. The advanced trends of SOS in the Cold zone, Temperate zone, and Tibetan Plateau zone exhibited accelerated, stalled, and reversed patterns after the corresponding TP, respectively. The TP did not occurred in Cold‐Temperate zone, where the SOS showed a consistent and continuous advance. TPs of EOS trends in the Cold zone, Cold‐Temperate zone, Temperate zone, and Tibetan Plateau zone occurred in the late 1980s or mid‐1990s. The EOS in the Cold zone, Cold‐Temperate zone, Temperate zone, and Tibetan Plateau zone showed weak advanced or delayed trends after the corresponding TP, which were comparable with the delayed trends before the corresponding TP. The shift patterns of LOS trends were primarily influenced by the shift patterns of SOS trends and were also heterogeneous within climatic zones.

## 1 INTRODUCTION

Phenology of surface vegetation is highly sensitive to regional and global climate change (Cleland, Chuine, Menzel, Mooney, & Schwartz, 2007; Fu et al., 2015; Menzel & Fabian, 1999; Menzel et al., 2006; Wang et al., 2016), and its interannual changes can strongly affect the carbon balance, as well as nitrogen and water cycles of global ecosystems (Cornelissen et al., 2007; Piao et al., 2015; Richardson et al., 2010; Shen et al., 2014). Changes in vegetation phenology are also mostly a direct and obvious reaction to the impact of global climate change on terrestrial ecosystems (Piao, Fang, Zhou, Ciais, & Zhu 2006; Zhao et al., 2015). Taking global warming into consideration, vegetation activities and vegetation phenology have significantly shifted at the global and regional scales (Fu et al., 2014; Liu, Fu, Zhu, et al., 2016a; Piao, Wang, et al., 2011a; Zhao et al., 2015). Therefore, the phenological changes in different scales are increasingly attracting the attention of global ecology and environmental researchers (Badeck et al., 2004; Cong et al., 2012; Schwartz, Ahas, & Aasa 2006).

Traditional ground‐based observations are widely used for the species level and for small‐scale areas because detailed information can be documented at the observation; however, these observations are limited by the location and number of observation sites and spatial scopes (Cleland et al., 2007; Studer, Stöckli, Appenzeller, & Vidale, 2007; Wu & Liu, 2013; Zhu et al., 2012). Remote sensing, in another way, provides continuously spatial and temporal informations on a variety of surface vegetation at regional or global scales, and numerous studies have investigated the variations of vegetation phenology in different regions over different time periods based on satellite‐measured Normalized Difference Vegetation Index (NDVI) datasets (Fu et al., 2014; Myneni, Keeling, Tucker, Asrar, & Nemani 1997; Shen et al., 2018; Wang et al., 2016; White et al., 2009; Wu & Liu, 2013; Zhang, Tarpley, & Sullivan 2007).

Most previous studies reported a consistent observation of a significant advanced trend in start date of vegetation growing season (SOS) at the middle and high latitudes of the Northern Hemisphere during the 1980s and 1990s (Chen, Hu, & Yu 2005; Myneni et al., 1997; Piao et al., 2006; Tucker et al., 2001; Zhou et al., 2001). With the accumulation of remote sensing data, some studies based on long‐term GIMMS NDVI dataset found a weakened, even delayed trend of SOS occurring since the late 1990s in many temperate regions of the Northern Hemisphere, such as East Asia, the Tibetan Plateau, and temperate China (Fu et al., 2014; Jeong, Ho, Gim, & Brown 2011; Piao, Cui, et al., 2011b; Wu & Liu, 2013; Yu, Luedeling, & Xu 2010). However, Wang et al. (2016) revealed that the SOS was advanced throughout the study period spanning from 1982 to 2012 in high latitudes of the Northern Hemisphere, from 55°N to 75°N. As the third pole of the Earth, the Tibetan Plateau is an opener and regulator of climate change in the Northern Hemisphere, and its SOS changes have always been the focus of debate in phenology studies. Based on the GIMMS NDVI during the period spanning 1982–2006, Yu et al. (2010) found that the advanced trend in SOS has significantly reversed after the mid‐1990s under increasing winter and spring temperatures in Tibetan Plateau. Subsequently, by integrating three different NDVI datasets, Zhang, Zhang, Dong, and Xiao (2013) revealed that the SOS in Tibetan Plateau showed a continuous advanced trend between 1982 and 2011. However, this result was later questioned by the study from Shen et al. (2013), who posited that the continuously advanced trend in SOS estimated by Zhang et al. (2013) is due to the lack of sufficient evidence and that the result is probably caused by not correcting for the changed pregrowth NDVI. In comparison to the debates on SOS change, the studies about the end date of vegetation growing season (EOS) were less reported, particularly on regional scales. EOS is also a critical variable in determining the length of vegetation growing season (LOS; Garonna et al., 2014) and regulates the balance of energy flow and material cycling in global and regional ecosystems (Estiarte & Peñuelas, 2015; Piao, Friedlingstein, Ciais, Viovy, & Demarty 2007; Richardson et al., 2013). A delayed trend in EOS during the past three decades has been detected at the global and the Northern Hemisphere scales (Garonna et al., 2014; Liu, Fu, Zhu, et al., 2016a; Zeng, Jia, & Epstein 2011), and the trend has weakened in several regions of the Northern Hemisphere during the 1990s and 2000s (Jeong et al., 2011; Yang, Guan, Shen, Liang, & Jiang 2015; Zhao et al., 2015). However, the shifting pattern of EOS trend is still largely unclear among those studies.

In this paper, we monitored the variations of vegetation phenology (SOS, EOS, and LOS) in different climatic zones in the middle and eastern Eurasia region from 1982 to 2015. The middle and eastern Eurasia region has a vast territory, covering a wide range of ecosystems and climates, and including rich biodiversity. In the study area, the climate change on the Tibetan Plateau has a great impact on the climate of the Eurasia and even the Northern Hemisphere (Li et al., 2003; Piao, Cui, et al., 2011b; Shen, 2011), and vegetation phenology in the Tibetan Plateau is highly sensitive to climate change (Yu et al., 2010). The boreal forest in the middle and eastern Eurasia region is less affected by human activities, and its vegetation phenology changes can accurately reflect the impact of climate change on forest ecosystems. In addition, change of vegetation phenology in the Cold zone of the Arctic Circle is important for indicating climate change in the polar regions. The great diversity of bio‐climate zones in middle and eastern Eurasia region provides a good opportunity for examining effects of climate change on vegetation phenology and their changes. Previous studies have made great efforts to detect the trend of vegetation phenology in the middle and eastern Eurasia region. However, researchers discovered inconsistent and even conflicting results concerning the response of phenology trends to climate change during different time periods and in different regions (Fu et al., 2014; Jeong et al., 2011; Piao et al., 2006; Shen et al., 2013; Wang et al., 2015; Wu & Liu, 2013; Yu et al., 2010; Zhang et al., 2013). Therefore, the trend of vegetation phenology and its response to climate change should be further investigated with the accumulation of satellite observation data. At present, a new generation of GIMMS NDVI 3g dataset has been extended to 2015 (Liu et al., 2018; Shen et al., 2018; Xia et al., 2018), and its quality is better than the previous version (Dai et al., 2018; Zhou et al., 2019). Longer‐term data are more useful for us to understand the relationship between phenology and climate change. We hypothesized that there are divergent responses of vegetation phenology to climate change in different regions related to the different climate backgrounds. Therefore, we applied the Polyfit‐Maximum (P‐M) method to estimate the SOS, EOS, and LOS in the middle and eastern Eurasia region using long‐term satellite NDVI data from 1982 to 2015. The aims of this study were (a) to quantify the long‐term trends of vegetation phenology for the entire study area and its four climatic zones, and (b) to clarify the shift patterns of phenology trends in different climatic zones.

## 2 MATERIALS AND METHODS

### 2.1 Study area

**Figure 1:**
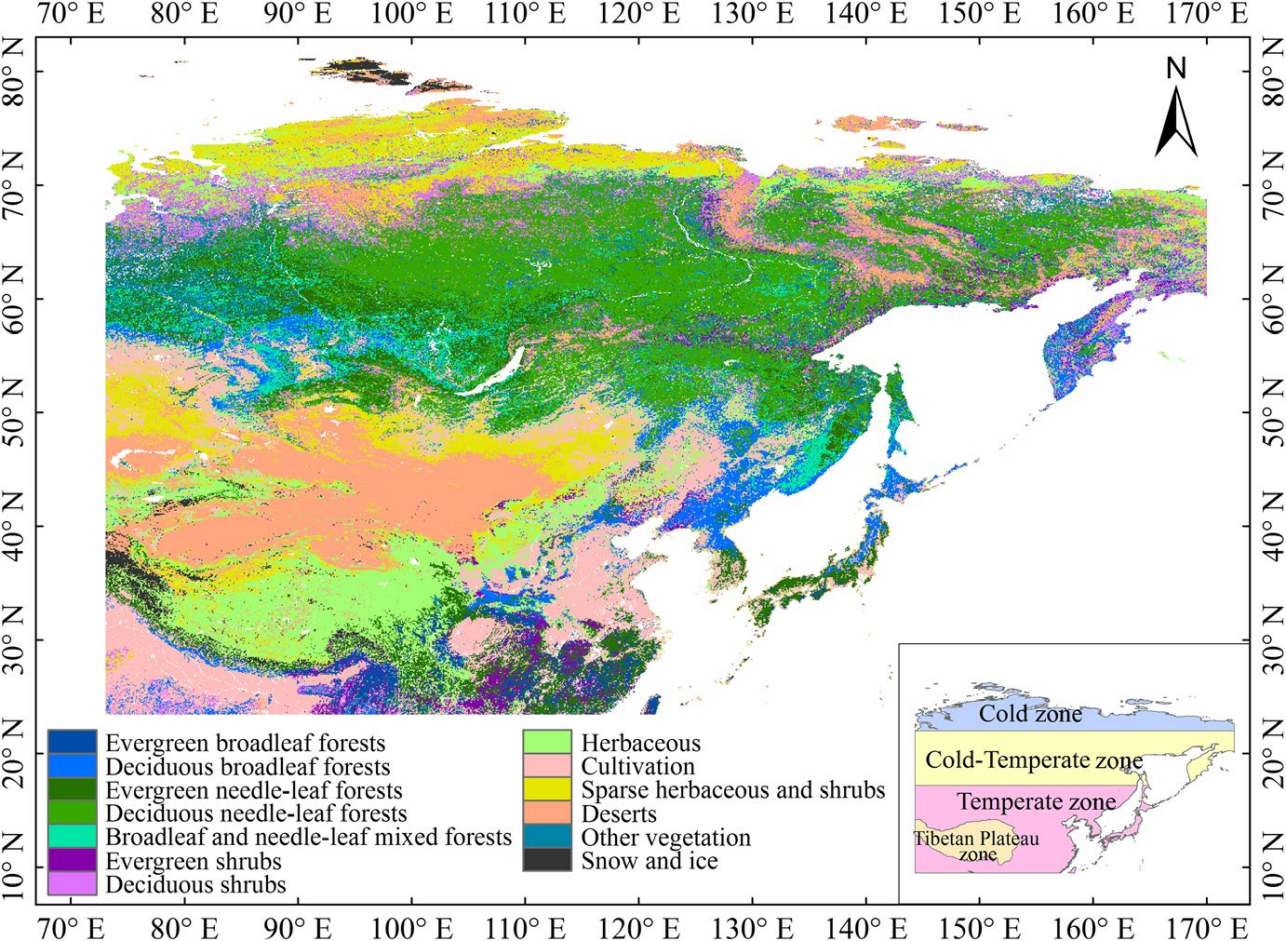
Location of the study area and four climatic zones as well as the spatial distribution of vegetation in the middle and eastern Eurasia region. The bottom right inset shows the location of the four climatic zones

We selected the middle and eastern Eurasia region (73°–170°E and 23.5°–85°N; Figure 1) as the study area, where seasonal changes of vegetation are clear and where the satellite‐derived NDVI dataset is least affected by the solar zenith angle in the middle and high latitudes of the Northern Hemisphere (Cong et al., 2013; Jeong et al., 2011; Piao et al., 2006; Slayback, Pinzon, Los, & Tucker 2003). Our study excluded the pixels of croplands because their vegetation phenology is intensively influenced by anthropogenic activities (Liu, Fu, Zeng, et al., 2016b; White, Thornton, & Running 1997; Zhang, Friedl, Schaaf, & Strahler 2004). Areas covered by evergreen vegetation were also excluded from our study because of the lack of obvious seasonality in surface greenness (Yang et al., 2015). Moreover, this study removed the pixels from nonvegetation areas, water bodies, and areas with an annual mean NDVI value during 1982–2015 that is smaller than 0.1 in order to reduce the influence of bare land and sparse vegetation on the interannual curve of NDVI (Jeong et al., 2011; Piao et al., 2006; Zhou et al., 2001).

To analyze the spatial pattern of phenology trends, the study area was divided into four climatic zones, including Cold zone (73°–170°E and 66.5°–85°N), Cold‐Temperate zone (73°–170°E and 50°–66.5°N), Temperate zone (73°–170°E and 23.5°–50°N), and Tibetan Plateau zone (73°–105°E and 26°–40°N; Figure 1). Cold zone is defined as the higher latitudes above the Arctic Circle, and representative vegetation is the tundra. Cold‐Temperate zone is defined as between the 50°N zone and the Arctic Circle, and representative vegetation is the boreal forest. Temperate zone is defined as between the Tropic of Cancer and 50°N. Boundary data of the Tibetan Plateau were obtained from the Third Pole Environment Database (http://www.tpedatabase.cn/portal/MetaDataList.jsp), and dominant vegetation is Alpine meadows and grasslands.

### 2.2 Data collection and generation

The study used the latest and longest release of GIMMS NDVI 3g version 1 dataset, which was provided by the Global Inventory Modeling and Mapping Studies (GIMMS) group based on remote sensing data obtained from NOAA/AVHRR series satellites. The NDVI 3g dataset, with a spatial resolution of 0.0833° and a temporal resolution of 15 days during the period spanning 1982 to 2015, were downloaded from the NASA Ames Ecological Forecasting Lab (https://ecocast.arc.nasa.gov/data/pub/gimms/3g.v1/). The long‐term NDVI dataset has been corrected to eliminate the contamination and noise caused by clouds, orbital drift, atmospheric interference, and the solar zenith angle (Cong et al., 2013; Piao et al., 2006; Pinzon & Tucker, 2014). The new generation of the NDVI dataset added percentile data and recovered NDVI negative values of snow‐covered regions in the winter at high latitudes in the Northern Hemisphere. The NDVI 3g dataset has been widely applied for the quantification of long‐term changes in vegetation growth (Piao et al., 2006; Shen et al., 2018; Wu & Liu, 2013; Xia et al., 2018; Yu et al., 2017; Yu, Liu, et al., 2013a).

Vegetation type data in our study were obtained from a land cover classification map, which was provided by the Joint Research Centre of the European Commission under the project of Global Land Cover 2000 (GLC2000; http://bioval.jrc.ec.europa.eu/products/glc2000/glc2000.php; Yang et al., 2015). The GLC2000 was made from the SPOT4\VEGETATION data by the Land Cover Classification System of Food and Agriculture Organization and included 22 land cover types (Bartholomé & Belward, 2005). The land classification map with a spatial resolution of 1 km was reprojected and resampled, to match NDVI 3g dataset.

### 2.3 Determining the SOS and EOS

Before detecting vegetation phenology, we removed the cloud and snow contaminations for each annual time series of NDVI data. First, we distinguished the pixels with potentially covered by cloud or snow using the flag data, which was calculated from the percentile data. These contaminated values were replaced by the temporally nearest valid NDVI values (Liu, Fu, Zhu, et al., 2016a). Second, the snow contamination was further minimized due to uncertainty of the flag data (Zhang et al., 2007). We screened out a time series of annual smallest NDVI values from 1982 to 2015 for each pixel. The median value of the smallest NDVI series in each pixel was extracted as surface background NDVI value, which was applied to replace the smaller values in annual NDVI time series (Zhang et al., 2007).

Previous studies have developed a variety of methods for extracting the SOS and EOS (Chen et al., 2004; Heumann, Seaquist, Eklundh, & Jönsson 2007; Markon, Fleming, & Binnian 1995; Piao et al., 2006; White et al., 2009, 1997; Yu, Price, Ellis, & Shi 2003). In our study, we adopted the P‐M method, which has been widely applied for the extraction of vegetation phenology in middle and high latitudes of the Northern Hemisphere (Fu et al., 2014; Jeong et al., 2011; Piao et al., 2006; White et al., 2009) to calculate the periods of greatest increase and decrease in the seasonal NDVI time series as SOS and EOS measurements (Jeong et al., 2011; Piao et al., 2006; White et al., 2009; Wu & Liu, 2013; Yang et al., 2015). First, the relative NDVI increase, NDVI_ratio_, was calculated from the 34‐year average (from 1982 to 2015) NDVI time curves with a temporal resolution of 15 days for each pixel using Equation (1):

(1) $${\text{NDVI}}_{\text{ratio}}=\frac{\text{NDVI}(t+1)-\text{NDVI}(t)}{\text{NDVI}(t)}$$

where *t* is time (temporal resolution of 15 days). Second, the NDVI(*t*), corresponding to the maximum NDVI_ratio_ at time *t*, is used as the threshold for detecting the SOS (Piao et al., 2006; Wu & Liu, 2013). The NDVI(*t* + 1) at time (*t* + 1), corresponding to the minimum NDVI_ratio_ at time *t*, is used as the threshold for detecting the EOS (Liu, Fu, Zeng, et al., 2016b; Piao et al., 2006; Yang et al., 2015). Finally, the NDVI time series data at 15 day intervals from January to September (for SOS) and from July to December (for EOS) for each year are fitted by a sixth‐degree polynomial function (Piao et al., 2006; Wu & Liu, 2013; Yang et al., 2015; Equation (2)), to reconstruct the NDVI time series with a temporal resolution of 1 day. The SOS and EOS are determined from the reconstructed daily NDVI time series curves based on the corresponding threshold for each pixel.

(2) $$\text{NDVI}=a_{0}+a_{1}x+a_{2}x^{2}+a_{3}x^{3}+\cdots +a_{n}x^{n}$$

where *x* is the day (Julian calendar). The regression coefficients (*a*_0_, *a*_1_, *a*_2_, *a*_3_…*a*_n_) are determined by the least square regression. In addition, the EOS subtracts the SOS of the corresponding pixel, as well as the LOS for each pixel.

### 2.4 Statistical analysis

The long‐term trends in SOS, EOS, and LOS for the entire study area and for the four climatic zones were estimated by linear regression during the period spanning 1982–2015. In addition, we applied a piecewise linear regression model (Toms & Lesperance, 2003; Equation (3)) to detect the potential turning point (TP) in the SOS, EOS, and LOS time series from 1982 to 2015 (Piao, Wang, et al., 2011a; Wang et al., 2011). However, we only detected the TP from the period of 1987–2010 in SOS, EOS, and LOS time series to avoid linear regressions containing too few data points in each period (Wang et al., 2011; Wu & Liu, 2013). A 3‐year moving average method was applied to smooth the SOS, EOS, and LOS time series before the piecewise linear regression application. The purpose is to eliminate statistical uncertainties due to the first and last data points and individual abnormalities in the phenological time series (Wu & Liu, 2013).

(3) $$y=\left\{\begin{array}{cc} \lambda_{0}+\lambda_{1}t+\varepsilon & t<\alpha \\ \lambda_{0}+\lambda_{1}t+\lambda_{2}\left(t-\alpha \right)+\varepsilon & t\geq \alpha \end{array}\right.$$

where *t* is year; *y* is value of the SOS, EOS, and LOS time series in each climatic zone and the entire study area; *α* represents the TP in the SOS, EOS, and LOS trends; *λ*_0_, *λ*_1_, and *λ*_2_ are regression coefficients; and *ε* is the residual random error. *λ*_1_ and *λ*_1_ + *λ*_2_ represent the piecewise linear trends (SOS, EOS, and LOS) before and after the TP. Least square regression is applied to determine *α* and other coefficients. To assess the necessity of introducing TP, a *t* test was used to verify the following null hypothesis: “*λ*_2_ is not significantly different from zero” (Wang et al., 2011; Wu & Liu, 2013). A *p* value <0.05 was considered to be significant. Using simple linear regression, we also calculated linear trends in the SOS, EOS, and LOS before and after the TP.

## 3 RESULTS

### 3.1 Trends in SOS and its discrepancies among different climatic zones

**Figure 2:**
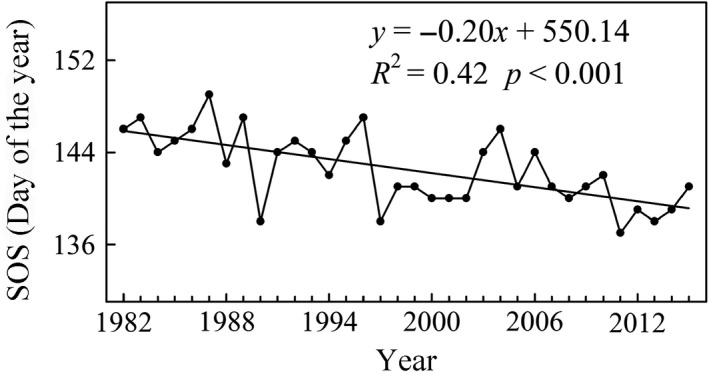
Interannual variations of SOS in the middle and eastern Eurasia region. The black solid line represents a linear fit during 1982–2015

**Figure 3:**
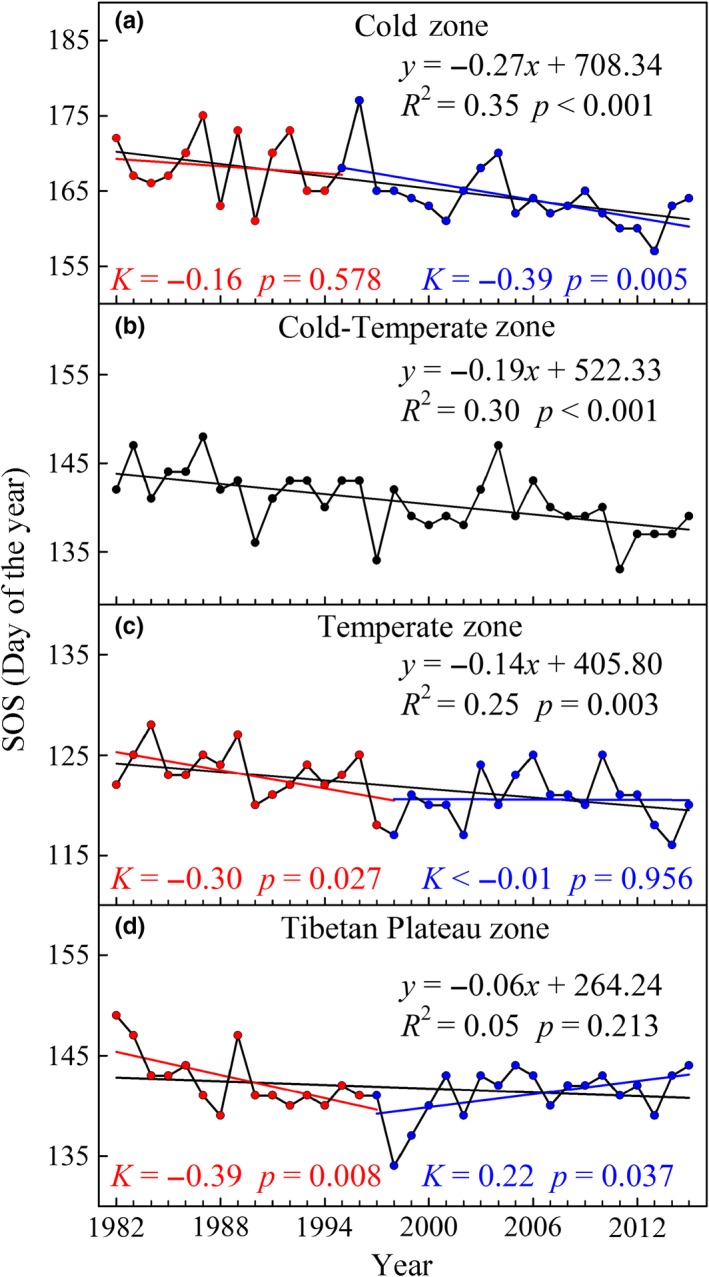
Interannual variations of SOS in four climatic zones from 1982 to 2015. (a) Cold zone, (b) Cold‐Temperate zone, (c) Temperate zone, and (d) Tibetan Plateau zone. The black solid line represents the linear fit during 1982–2015, the red solid line represents the linear fit before TP, and the blue solid line represents the linear fit after TP

The mean SOS in the middle and eastern Eurasia region experienced a significant advance from 1982 to 2015 (*R* ^2^ = 0.42, *p* < 0.001), which was best described by a linear trend with a rate of 0.20 days/year (Figure 2). The average SOS happened on calendar day 143 (Julian calendar) over the past 34 years. Additionally, three earliest SOS was found in 1990, 1997, and 2011. The rates of SOS trends had large variations among different climatic zones, although all of them exhibited an advanced trend during the period from 1982 to 2015 (Figure 3). The Cold zone had the largest and significantly advanced trend during the same period with a rate of 0.27 days/year (*p* < 0.001; Figure 3a), whereas the Tibetan Plateau zone did not show a significant trend (*p* = 0.213; Figure 3d). Both advanced rates of SOS in the Cold‐Temperate zone and Temperate zone were 0.19 days/year (*p* < 0.001) and 0.14 days/year (*p* = 0.003), respectively (Figure 3b,c).

The TPs of SOS trends in the Cold zone, Temperate zone, and Tibetan Plateau zone occurred in 1995, 1998, and 1997 (Table 1), respectively. To further explore the shift patterns of SOS trends, we calculated the linear trends in SOS for the three climatic zones during the two periods before and after the corresponding TP. The SOS in three climatic zones had experienced a consistent advanced trend during the period before the corresponding TP, and the advanced rates of SOS in the Cold zone, Temperate zone, and Tibetan Plateau zone were 0.16 days/year (*p* = 0.578), 0.30 days/year (*p* = 0.027), and 0.39 days/year (*p* = 0.008), respectively (Figure 3a,c,d). However, the three climatic zones showed different trends during the period after the corresponding TP. The SOS advance in the Cold zone accelerated significantly with an average rate of 0.39 days/year (*p* = 0.005), whereas Temperate zone exhibited a stalled state (*K* < −0.01 days/year, *p* = 0.956; Figure 3a,c). Furthermore, the Tibetan Plateau zone showed a delayed trend with a rate of 0.22 days/year (*p* = 0.037) in the later period (Figure 3d).

**Table 1:** TPs in the SOS, EOS and LOS trends during 1982–2015

Climatic zone	SOS	EOS	LOS
Cold zone	1995*	1995***	NO
Cold‐temperate zone	NO	1987***	1989*
Temperate zone	1998**	1994***	1997***
Tibetan plateau zone	1997***	1989**	1991***

Abbreviation: NO: no turning point.

* 0.05 > *p* > 0.01.

** 0.01 > *p* > 0.001.

*** *p* < 0.001.

### 3.2 Trends in EOS and its discrepancies among different climatic zones

**Figure 4:**
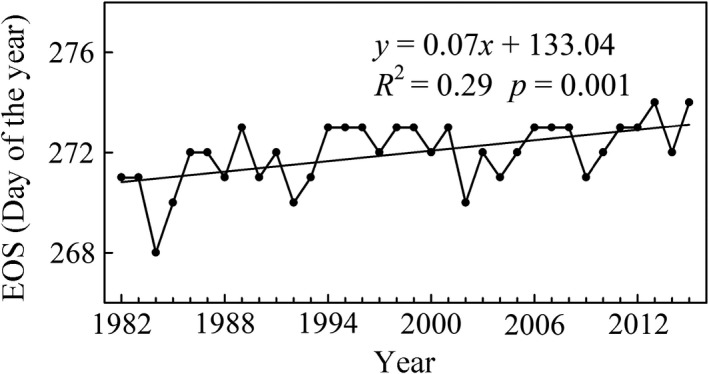
Interannual variations of EOS in the middle and eastern Eurasia region. The black solid line represents the linear fit during 1982–2015

**Figure 5:**
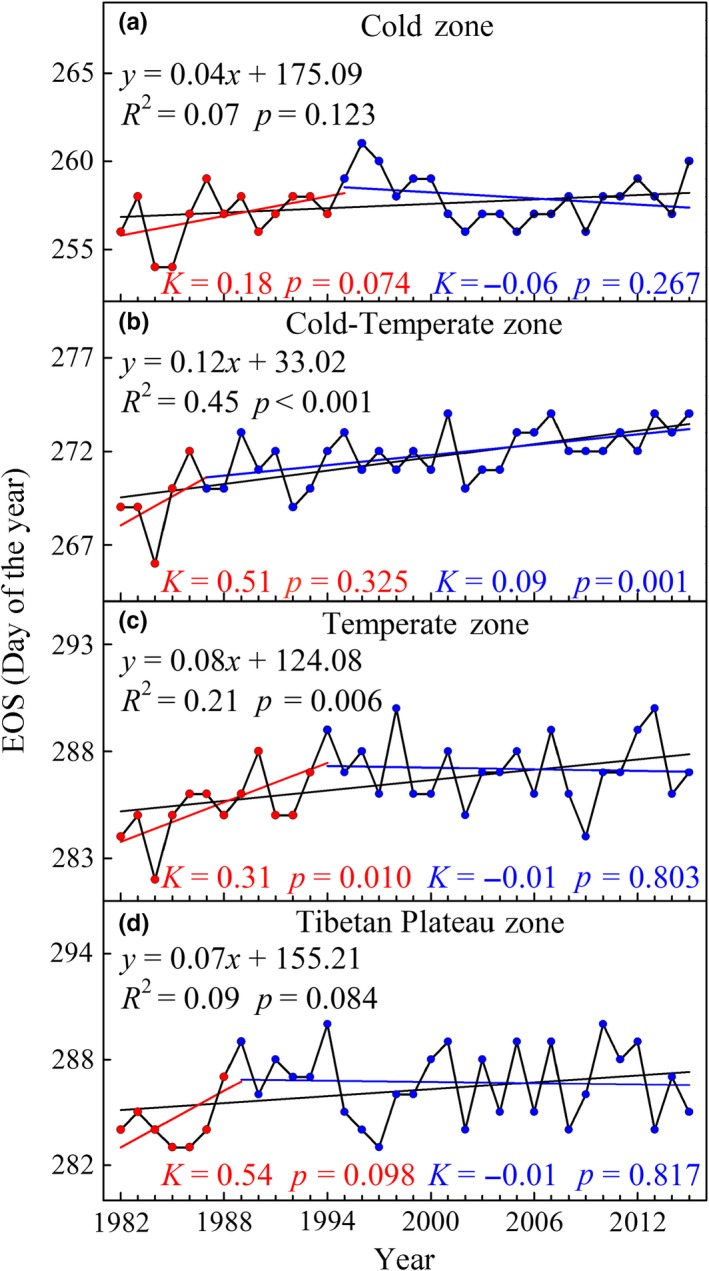
Interannual variations of EOS in four climatic zones from 1982 to 2015. (a) Cold zone, (b) Cold‐Temperate zone, (c) Temperate zone, and (d) Tibetan Plateau zone. The black solid line represents the linear fit during 1982–2015; the red solid line represents the linear fit before TP; and the blue solid line represents the linear fit after TP

The mean EOS during 1982–2015 over the entire study area showed a slow delayed trend with an average rate of 0.07 days/year (*R* ^2^ = 0.29, *p* = 0.001; Figure 4). The EOS changed from day 270 (Julian calendar) in the early 1980s (average of 1982–1985) to day 273 (Julian calendar) in the early 2010s (average of 2011–2015), which further illustrated that the EOS changes were minimal in the entire region. During 1982–2015, the EOS in the Cold‐Temperate zone and Temperate zone experienced a significantly delayed trend (*p* < 0.05), whereas the EOS in the Cold zone and Tibetan Plateau zone showed an insignificant delayed trend (*p* > 0.05; Figure 5). The largest delay in EOS over the past 34 years occurred in the Cold‐Temperate zone (0.12 days/year, *p* < 0.001) followed by the Temperate zone (0.08 days/year, *p* = 0.006), Tibetan Plateau zone (0.07 days/year, *p* = 0.084), and Cold zone (0.04 days/year, *p* = 0.123; Figure 5).

The result of TP extraction from EOS trends showed that the TP in the Cold‐Temperate zone and Tibetan Plateau zone occurred in 1987 and 1989, whereas the TP in the Cold zone and Temperate zone occurred in 1995 and 1994 (Table 1). Upon further analysis of the linear trends during the two periods before and after the corresponding TP, we found that the delayed trends of EOS in the four climatic zones were stalled and even reversed during the later period. Except for a significant delayed trend in the Temperate zone (*K* = 0.31 days/year, *p* = 0.010; Figure 5c), the EOS trends in the Cold zone, Cold‐Temperate zone, and Tibetan Plateau zone during the period before the corresponding TP experienced an insignificant delay by rates of 0.18 days/year (*p* = 0.074), 0.51 days/year (*p* = 0.325), 0.54 days/year (*p* = 0.098), respectively (Figure 5a,b,d). The EOS in the Cold zone, Temperate zone, and Tibetan Plateau zone during the period after the corresponding TP all exhibited an insignificantly advanced trend (Figure 5a,c,d). However, the EOS in the Cold‐Temperate zone had a delay after the corresponding TP by a rate of 0.09 days/year (*p* = 0.001; Figure 5b).

### 3.3 Trends in LOS and its discrepancies among different climatic zones

**Figure 6:**
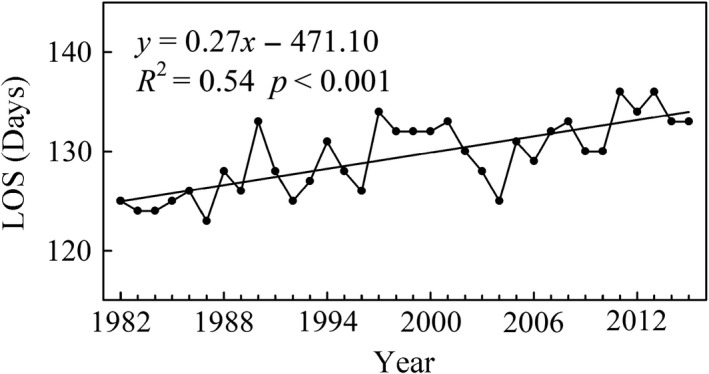
Interannual variations of LOS in the middle and eastern Eurasia region. The black solid line represents the linear fit during 1982–2015

**Figure 7:**
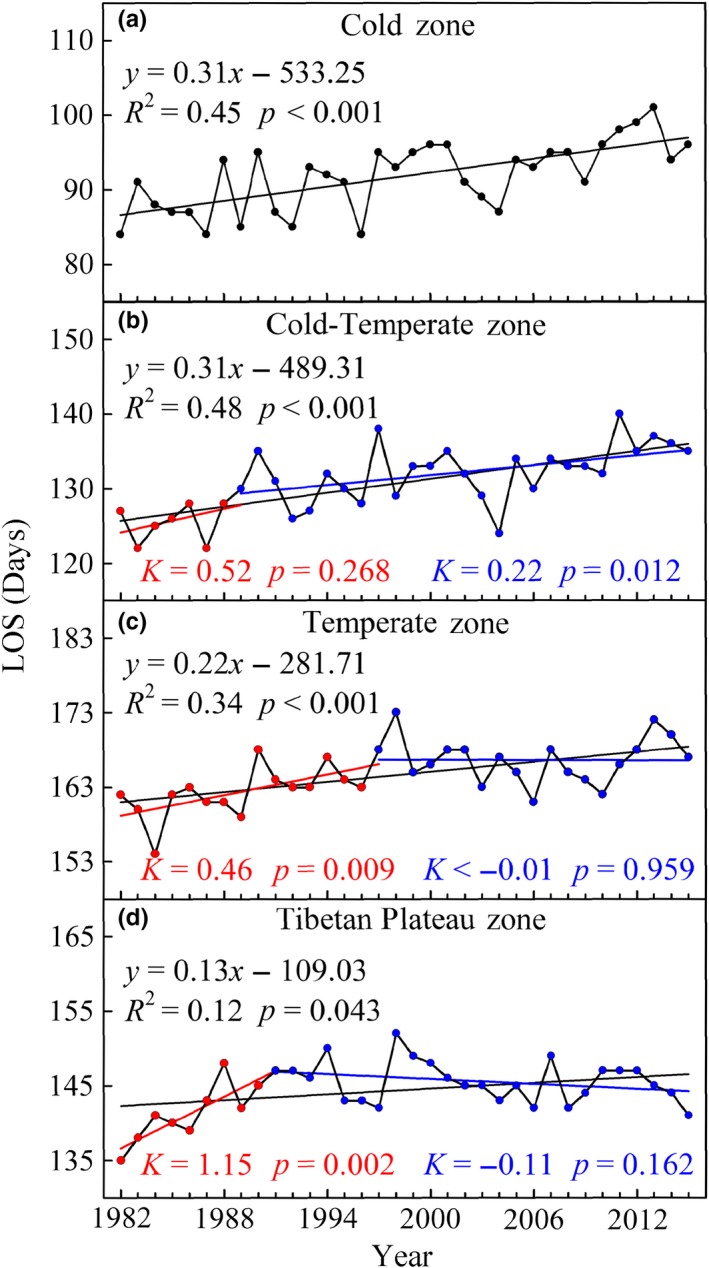
Interannual variations of LOS in four climatic zones from 1982 to 2015. (a) Cold zone, (b) Cold‐Temperate zone, (c) Temperate zone, and (d) Tibetan Plateau zone. The black solid line represents the linear fit during 1982–2015, the red solid line represents the linear fit before TP, and the blue solid line represents the linear fit after TP

The mean LOS over the middle and eastern Eurasia region significantly increased (*R* ^2^ = 0.54, *p* < 0.001) by a rate of 0.27 days/year over the past 34 years (Figure 6). Furthermore, the longest LOS occurred in 2011 and 2013 with a length of 136 days. The extended trends were found in the four climatic zones. The rates of LOS extension in the Cold zone, Cold‐Temperate zone, Temperate zone, and Tibetan Plateau zone through the entire period of 1982–2015 were 0.31 days/year (*p* < 0.001), 0.31 days/year (*p* < 0.001), 0.22 days/year (*p* < 0.001), and 0.13 days/year (*p* = 0.043), respectively, and the trends were significant (Figure 7).

TPs were also detected in the Cold‐Temperate zone, Temperate zone, and Tibetan Plateau zone, and they occurred in 1989, 1997, and 1991 (Table 1), respectively. LOS in the Temperate zone and Tibetan Plateau zone were significantly extended before the corresponding TP (*K* = 0.46 days/year, *p* = 0.009; *K* = 1.15 days/year, *p* = 0.002, respectively; Figure 7c,d), but the LOS trends were reversed in both climatic zones after the corresponding TP, particularly in the Tibetan Plateau zone where the LOS showed a clearly shortened trend (*K* = −0.11 days/year, *p* = 0.162; Figure 7d). For the Cold‐Temperate zone, the extended trend during 1982–1989 (*K* = 0.52 days/year, *p* = 0.268) decreased during 1989–2015 (*K* = 0.22 days/year, *p* = 0.012; Figure 7b), whereas trend in the Cold zone was consistent and significant over the entire period (*K* = 0.31 days/year, *p* < 0.001; Figure 7a).

## 4 DISCUSSION

### 4.1 Changes in SOS trends

The mean SOS during the period spanning 1982–2015 over the middle and eastern Eurasia region revealed an overall significantly advanced trend (−0.20 days/year, *p* < 0.001; Figure 2). The trend is close to −0.17 ± 0.06 days/year in Eurasia from 1982 to 2011 reported by Wang et al. (2015). Compared with other regions in the middle and high latitudes of the Northern Hemisphere, the estimation of SOS trend in our study is smaller than that from Fu et al. (2014), who detected an advanced trend by 0.45 days/year in Western Central Europe from 1982 to 2011. However, the SOS trend in the middle and eastern Eurasia region, exhibiting an advanced rate of 0.19 days/year (during 1982–2008), is larger than the result reported by Zeng et al. (2011), who estimated an advanced trend with a rate of 0.03 days/year in North America from 1982 to 2008. In addition, White et al. (2009) found no significant advanced trend in SOS from 1982 to 2006 in North America. This finding indicates that the SOS trend in Eurasia is larger than that in North America and less than that in Europe. The result is consistent with the conclusions of Jeong et al. (2011) and Barichivich et al. (2013). This finding is considered to be probably associated with spatial asymmetry of spring temperatures increasing in the Northern Hemisphere because spring phenology is a sensitive indicator of the response of terrestrial ecosystems to climate change (Cohen, Furtado, Barlow, Alexeev, & Cherry 2012; Menzel & Fabian, 1999; Menzel et al., 2006; Schwartz, 1998). Therefore, the regional differences of SOS trends should be further discussed on the condition that vegetation responses are associated with climate factors. Long‐term trends of SOS in the four climatic zones illustrated that the higher latitude areas had a higher advanced rate, which is consistent with the finding of Post, Steinman, and Mann (2018), who also found rapidly advanced trend in SOS at higher latitudes of the Northern Hemisphere. Previous studies revealed that the advance in SOS was related to preseason temperature warming (Chen et al., 2005; Myneni et al., 1997; Piao et al., 2006) and that warming in higher latitudes of the Northern Hemisphere was stronger than that in lower latitudes (Hassol, 2004; Post et al., 2018). Warmer springs could easily thaw the topsoil and bring earlier snow melts and lead to germination of early spring vegetation (Parmesan & Yohe, 2003; Richardson, Bailey, Denny, Martin, & O'Keefe, 2006; Yu et al., 2010).

Linear analyses showed that the advanced trend in SOS was significantly reversed after 1997 in the Tibetan Plateau zone, whereas the SOS advance was stalled after 1998 in the Temperate zone. Similarly, Jeong et al. (2011) showed that the earlier SOS in the temperate Northern Hemisphere has weakened since the mid‐1990s, and Piao, Cui, et al. (2011b) revealed that the advanced trend in SOS appeared to be reversed after the late 1990s in the Tibetan Plateau zone. However, the SOS in the Cold zone showed an accelerated advance after the corresponding TP, and the SOS in the Cold‐Temperate zone showed a continuously advanced trend during the entire 34 years. Overall, the shift patterns of SOS trends in the middle and eastern Eurasia region are spatially heterogeneous, and the four climatic zones exhibited four shift patterns of SOS trends. Studies indicated that spring warming advanced the SOS in most regions of the Northern Hemisphere (Chen et al., 2005; Cong et al., 2013; Menzel et al., 2006; Piao et al., 2006). The weakened advance of SOS in the Temperate zone could be explained by the reversal or stalling of spring temperatures since the late 1990s (Jeong et al., 2011; Wang et al., 2011; Wu & Liu, 2013). Previous studies showed that global warming trends have decelerated over the past decade (Cane, 2010; Chen & Tung, 2014; Solomon et al., 2010). In contrast, warming in the spring at higher latitudes of the Northern Hemisphere has lasted over the recent decades (Kaufman, Schneider, McKay, Ammann, & Bradley, 2009; Li, Wang, Zhang, Li, & Zang, 2017; Serreze & Barry, 2011). Therefore, the SOS in Cold zone and Cold‐Temperate zone still significantly advanced after the mid‐1990s. In addition, the tundra vegetation in Cold zone is more sensitive to rises in temperature (Chapin, Eugster, Mcfadden, Lynch, & Walker, 2000; Hinzman et al., 2005).

Compared with other climatic zones, the reversed trend of SOS in the Tibetan Plateau zone is a unique phenomenon in Eurasia. Although the Tibetan Plateau zone with its lower annual mean temperatures is similar to the Cold‐Temperate zone, and while the vegetation in the Tibetan Plateau zone is similar to the Cold zone with the predominance of tundra vegetation, the Tibetan Plateau zone shows a very different shift pattern of spring phenology. Yu et al. (2010) concluded that rapid warming in the winter induced a lack of chilling in the Tibetan Plateau zone, which could lead to a delay in SOS. Zhang et al. (2007) also found that chilling accumulations in the winter decreased due to the reduction of chilling days and, as a result, that SOS was delayed starting at 40°N and moving southward in North America. However, this explanation of the delay in SOS was later questioned by the following studies. Chen, Zhu, Wu, Wang, and Peng (2011) believed that the combined effect of the thawing‐freezing process, grassland degradation, and climate changes, but not the unitary effect of spring and winter warming, delayed the SOS. However, a recent study from Shen, Piao, Cong, Zhang, and Jassens (2015) found that the delay in SOS mainly occurred in the southwestern region of the Tibetan Plateau zone and was due to the decrease in spring precipitation. The Tibetan Plateau zone has a special plateau climate and is strongly influenced by the East Asia monsoon events. A strong 1997/98 El Nino event significantly advanced the SOS in the Tibetan Plateau zone in 1998 (Klein et al., 2014; Piao et al., 2006), and the SOS began to delay after 1998 despite the continued warming in winter and spring. Therefore, climate change and other factors (e.g., human activity) in the Tibetan Plateau zone can affect the changes in SOS. All of these issues require further study from different perspectives, such as dividing the Tibetan Plateau zone into multiple subunits, paying more attention to precipitation in the western part and strengthening the combination of ground observations and remote sensing observations.

### 4.2 Changes in EOS trends

Our study estimated that the EOS over the middle and eastern Eurasia region was delayed on average by a rate of 0.07 days/year from 1982 to 2015. Previous studies also documented that the EOS experienced a delayed trend in regions of the Northern Hemisphere, such as North America (Zhu et al., 2012), Eurasia (Li et al., 2017), Europe (Garonna et al., 2014; Stöckli & Vidale, 2004), temperate China (Liu, Fu, Zeng, et al., 2016b; Yang et al., 2015), and the Tibetan Plateau (Cong, Shen, & Piao 2017; Jin, Zhuang, He, Luo, & Shi 2013). Compared with other regions around the Northern Hemisphere at the same period, the delayed trend in EOS (0.07 days/year) is smaller than that in North America (0.36 days/year from 1982 to 2006; Zhu et al., 2012) and is also smaller than that in Europe (0.14 days/year compared to 0.47 days/year from 1982 to 2000; Stöckli & Vidale, 2004). Jeong et al. (2011) also discovered that the delayed trend of EOS in eastern Asia is smaller than that in the United States and Europe during 1982–2008. This finding revealed that there were obvious regional differences of EOS trends in the Northern Hemisphere. It may be related to asynchronous changes in summer and autumn temperatures between different regions (Cohen et al., 2012). Previous studies have proven that autumn phenology is severely affected by summer and autumn temperature (Che et al., 2014; Piao et al., 2006; Yang et al., 2015). On climatic zone scales, our estimation of EOS trend in the Tibetan Plateau zone (0.09 days/year, from 1982 to 2011) is closer to the result of Cong et al. (2017), who calculated a delay with a rate of 0.07 days/year over the past three decades. The EOS delay in the Temperate zone (0.08 days/year) is smaller than the result reported by Yang et al. (2015), who detected a delayed trend in EOS by 0.13 days/year over temperate China from 1982 to 2010. Similarly, the EOS trend in the Temperate zone (0.24 days/year, from 1982 to 1999) is also less than the estimation of Piao et al. (2006), who reported a delayed trend in EOS by a rate of 0.37 days/year over temperate China from 1982 to 1999. The rates of EOS trends had large discrepancies among regions and studies, which could be mainly due to differences in both study periods and areas.

Changes in EOS were not temporally consistent over the whole period. The delayed trends of EOS in the Cold zone and Temperate zone have reversed since the mid‐1990s, whereas the delayed trends of EOS in the Cold‐Temperate zone and Tibetan Plateau zone have slowed and reversed since the late 1980s. All the findings suggested that the delay in EOS disappeared or weakened in the middle and eastern Eurasia region over the last two decades. Previous studies also discovered that the delayed trends of EOS over temperate China had disappeared since the mid‐1990s (Yang et al., 2015). Studies suggested that warmer preseason (summer and autumn) temperatures delayed the EOS (Liu, Fu, Zhu, et al., 2016a; Piao et al., 2006; Yang et al., 2015). The preseason warming trends decelerated or even reversed in recent decades (Cane, 2010; Guemas, Doblas‐Reyes, Andreu‐Burillo, & Asif 2013; Kaufmann, Kauppi, Mann, & Stock, 2011; Yu, Sun, Liu, Wang, & Everman, 2013b), and the result could explain the shifting pattern of disappearing or reversing delayed trends in EOS.

### 4.3 Changes in LOS trends

LOS in the middle and eastern Eurasia region has significantly increased over the entire period by an average rate of 0.27 days/year. Previous studies based on NDVI data also showed an increased LOS in the Northern Hemisphere. For example, the LOS trend with an increasing rate of 0.36 days/year over the Northern Hemisphere from 1982 to 2008 was evaluated by Jeong et al. (2011). In addition, Wang et al. (2016) also reported that most regions of the Northern Hemisphere during the period from 1982 to 2012 experienced an increase in LOS. However, discrepancies in the rates of LOS increase existed among different continents or regions. The LOS in North America from 1982 to 2006 has significantly increased by a rate of 0.31 days/year (Zhu et al., 2012). In our study, the trend in LOS from 1982 to 2006 in the middle and eastern Eurasia region increased with a rate of 0.25 days/year. By analyzing MODIS and AVHRR data, a study from Zeng et al. (2011) showed that Arctic, Eurasian, and American regions had different rates of LOS increase.

Advanced SOS and delayed EOS lengthened LOS in the middle and eastern Eurasia region over the past 34 years. However, the advanced rates of SOS in the entire study area and over the four climatic zones were higher than the delayed rates of EOS, except for the Tibetan Plateau zone. This result is consistent with the conclusion of Zhao et al. (2015), who reported that the advanced rate of SOS was higher than that of EOS in Asia. In addition, similar to the variations of SOS trends, LOS trends also varied among different climatic zones, and the extended rates increased with increasing latitude. This revealed that the SOS is a key factor that regulates the LOS changes in middle and eastern Eurasia over the past 34 years. However, Zhu et al. (2012) concluded that the LOS was prolonged mainly due to the delay in EOS in North America during the period spanning from 1982 to 2006.

The increased rate of LOS slowed down in the Cold‐Temperate zone during the period after the corresponding TP, whereas it was reversed in the Tibetan Plateau zone. The increased trend of LOS in the Temperate zone disappeared after the mid‐1990s. The shift patterns of LOS trends in some regions of the Northern Hemisphere have been observed in previous studies (Jeong et al., 2011). This result was primarily due to the decrease and reversal of SOS trends or to the decrease and advance of the EOS trends during the period after the corresponding TP (Zhao et al., 2015).

## 5 CONCLUSIONS

In this study, we investigated the trend characteristics of SOS, EOS, and LOS in the middle and eastern Eurasia. From 1982 to 2015, the LOS has prolonged, mostly through an earlier SOS and a later EOS in the entire study area and each climatic zone. The SOS, EOS, and LOS trends in the four climatic zones in the middle and eastern Eurasia are not continuous throughout the 34‐year period. The SOS in different climatic zones had different shift patterns in response to the climate change over the past 34 years. The advanced trends of SOS in Cold zone, Temperate zone, and Tibetan Plateau zone showed accelerated, stagnant, and reversal patterns after the mid‐ or late 1990s, respectively. The delayed trends of EOS in the Cold zone, Cold‐Temperate zone, Temperate zone, and Tibetan Plateau zone slowed down or reversed after the late 1980s or mid‐1990s. The EOS delay disappeared or weakened in the middle and eastern Eurasia region over the last two decades. LOS changes and its trends are affected by SOS and EOS, but SOS is the dominant factor. Discrepancies in trend patterns of vegetation phenology reflect the spatial heterogeneity of climate change.

## CONFLICT OF INTEREST

None declared.

## AUTHOR CONTRIBUTIONS

Yuandong Zhang and Fengxue Gu conceived the ideas and designed the study. Yaobin Li downloaded and analyzed the experimental data. Yuandong Zhang and Yaobin Li drafted the manuscript. Fengxue Gu and Shirong Liu critically revised the manuscript. All authors approved the final manuscript.

## Data Availability

Data are available from Dryad Digital Repository, and DOI number is https://doi.org/10.5061/dryad.1498h4m.
